# Inferences of Diplodocoid (Sauropoda: Dinosauria) Feeding Behavior from Snout Shape and Microwear Analyses

**DOI:** 10.1371/journal.pone.0018304

**Published:** 2011-04-06

**Authors:** John A. Whitlock

**Affiliations:** Museum of Paleontology, University of Michigan, Ann Arbor, Michigan, United States of America; Raymond M. Alf Museum of Paleontology, United States of America

## Abstract

**Background:**

As gigantic herbivores, sauropod dinosaurs were among the most important members of Mesozoic communities. Understanding their ecology is fundamental to developing a complete picture of Jurassic and Cretaceous food webs. One group of sauropods in particular, Diplodocoidea, has long been a source of debate with regard to what and how they ate. Because of their long lineage duration (Late Jurassic-Late Cretaceous) and cosmopolitan distribution, diplodocoids formed important parts of multiple ecosystems. Additionally, fortuitous preservation of a large proportion of cranial elements makes them an ideal clade in which to examine feeding behavior.

**Methodology/Principal Findings:**

Hypotheses of various browsing behaviors (selective and nonselective browsing at ground-height, mid-height, or in the upper canopy) were examined using snout shape (square vs. round) and dental microwear. The square snouts, large proportion of pits, and fine subparallel scratches in *Apatosaurus*, *Diplodocus*, *Nigersaurus*, and *Rebbachisaurus* suggest ground-height nonselective browsing; the narrow snouts of *Dicraeosaurus*, *Suuwassea*, and *Tornieria* and the coarse scratches and gouges on the teeth of *Dicraeosaurus* suggest mid-height selective browsing in those taxa. Comparison with outgroups (*Camarasaurus* and *Brachiosaurus*) reinforces the inferences of ground- and mid-height browsing and the existence of both non-selective and selective browsing behaviors in diplodocoids.

**Conclusions/Significance:**

These results reaffirm previous work suggesting the presence of diverse feeding strategies in sauropods and provide solid evidence for two different feeding behaviors in Diplodocoidea. These feeding behaviors can subsequently be tied to paleoecology, such that non-selective, ground-height behaviors are restricted to open, savanna-type environments. Selective browsing behaviors are known from multiple sauropod clades and were practiced in multiple environments.

## Introduction

Herbivory evolved multiple times within Archosauria, the group uniting crocodiles and birds and containing a variety of fossil forms such as dinosaurs, aetosaurs, phytosaurs, rauisuchians, and pterosaurs. Among living archosaurs, however, herbivory is restricted to birds and potentially turtles, which have been recovered as the sister clade to crown-group Archosauria [1]–[3]. Jaw morphology in these living representatives is highly derived (e.g., secondary loss of teeth) and provides a poor analog for the vast majority of extinct, herbivorous archosaurs. Without a direct behavioral analog, progress in understanding the behavior of herbivorous archosaurs has not reached the level of sophistication seen in studies of extinct mammals, whose craniodental anatomy is readily interpreted using modern analogues and dental wear features. As a result, studies of Mesozoic ecology are often hampered by an incomplete understanding of herbivore behavior—an issue that carries great weight due to the relative abundance of herbivores in any ecosystem. As a major link between primary productivity and secondary and tertiary consumers, herbivores represent the base of the animal food pyramid and therefore influence the flow of energy through an ecosystem.

Of particular importance to the understanding of Mesozoic ecology are sauropod dinosaurs, which were the dominant megaherbivores during most of the Jurassic and Cretaceous, a span of approximately 135 million years. Sauropods were typically quite large, with the largest reaching over 30 m in total body length [4]. Although estimates of metabolic rates and food requirements vary [5]–[7], it is clear that these organisms required a large amount of browse daily. There is also evidence of herding behavior [8], increasing the local impact of sauropods on a community—higher population density results in greater stress on plant communities as more animals utilize the available resources. Disregarding all other impacts, these animals must have had major effects on communities in terms of bulk mass consumed daily.

Although sauropods lack the obvious adaptations for herbivory (e.g., beaks and cheeks) present in the other major clade of herbivorous dinosaurs, Ornithischia [9], ongoing work suggests that sauropods were equally specialized for the task. Histological study of thin-sectioned teeth reveals that sauropod dinosaurs had the fastest known tooth replacement rates among vertebrates—some sauropods replaced their teeth every 30 days [10]. Even the slowest replacing teeth in neosauropods (∼62 days; [11]) were replaced at a rate similar to the fastest replacement rate seen in non-sauropod dinosaurs [12]. Given the remarkable degree of wear seen on shed teeth—perhaps 25–40% crown height lost, based on estimated *Nigersaurus* crown heights [13]—the implication is that sauropod teeth experienced extreme wear throughout their short life span. As the plant materials a sauropod would have encountered are not particularly abrasive in comparison to modern floras [9], it is likely that a combination of feeding behavior and sheer volume of plant material ingested is responsible for high wear. Debate continues, however, on how and what sauropods, particularly diplodocoid sauropods, were eating. In this contribution, I examine the diet and behavior of diplodocid sauropods, using data from analyses of snout shape and dental wear (both micro- and macrowear features).

### Diplodocoid diets: previous studies

Within Sauropoda, no clade has received as much attention with regard to diet as Diplodocoidea [10], [14]–[33]. This is due in large part to their unusual skulls, which feature elongate, horse-like faces ending in a squared snout and a comparably short arcade of tiny, narrow-crowned teeth [34]. Because this morphology has typically been regarded as poorly suited for biting or slicing through vegetation, many other hypotheses of feeding behavior have been put forth, including eating plants from riverbeds [14], scraping algae from rocks[16], stripping leaves from branches by using the teeth as a sort of ‘comb’ or ‘rake’ [19], [20], [24], and at least partial reliance on carnivory, either on bivalves (Sternfeld in [15]) or fish [18]. Other workers have suggested that diplodocoid sauropods fed in a manner similar to modern large-bodied herbivores: ground-level browsing [10], [24], [27], . This latter hypothesis has the benefit of being directly observable in modern animals and thus requires no invocation of novel behaviors. Testing this hypothesis is consequently more straightforward and can be accomplished using methods proven effective in studies of both mammals and dinosaurs.

### Reconstructing diets

The reconstruction of diet in fossil taxa has a long methodological history, and in sauropods dates back to the 19th century [33]. Two morphological features in particular, snout shape and tooth wear, have proven informative in mammals. Here, I discuss methods historically used to examine those features, and their relevance to the reconstruction of diets in diplodocoid sauropods.

#### Snout shape

The shape of the premaxilla, constituting the entirety of the anterior-most extremity of the skull, or ‘snout’, in most mammals, has long been suggested to be related to dietary preference or selectivity in herbivorous mammals [35]–[38]. Boué [35] provided the first quantitative measurement of snout shape in ungulates, the Arcade Index (AI). The AI measured the shape of the incisive arcade in the dentary in ruminants and was calculated by dividing the breadth of the arcade by its anteroposterior depth. An AI of over 1.0 (a square jaw) was found to be associated with grazers, whereas scores below 1.0 (pointed jaws) were found most commonly in browsers, although this measurement is primarily a measure of breadth and does not fully capture snout shape.

Janis and Erhardt [39] found that although both palatal breadth and snout breadth scale with body size, palatal breadth was more strongly correlated with dietary selectivity (e.g., grazing vs. browsing behavior) in ungulates. The narrowest snout breadths occurred in animals browsing in upper story vegetation [39]. Gordon and Illius [40] expanded on these observations, noting that incisor arcade structure is also correlated with selectivity. Broader arcades are maladaptive for selective browsing behavior, as this morphology is more likely to result in the unintentional ingestion of unpalatable, undigestible, or dangerous woody parts of browse plants (e.g., thorns). Narrow arcades, conversely, are maladaptive for grazing (a non-selective feeding behavior) because the small breadth of the arcade results in reduced intake efficiency when consuming sward-like growth forms such as grasses. Gordon and Illius [40] also noted that grazers, in addition to having broader snouts, also have sublinear arcades with a more transversely oriented anterior tooth row. So-called ‘intermediate’ feeders, which may utilize both selective and non-selective behaviors, have snout shapes more similar to those of browsers than grazers. Gordon and Illius [40] concluded that snout shapes have evolved to maximize food intake within a particular nutritional quality constraint. There also appears to be a size component to behavioral and morphological differentiation: larger animals were found to be less selective than smaller taxa, although at body sizes below about 100 kg, morphological differentiation between snouts seems to be minor [40].

Solounias et al. [41] and Solounias and Moelleken [42] demonstrated that a similar relationship between snout shape and diet also occurred in extinct ungulates. These two studies used a method originally applied to hominids [43]. Snout shape was quantified along a profile defined using the midline and the intersection of the snout with a line drawn at 26° from the midline, originating at the anterior-most point of the premaxillary symphysis. These profiles were then digitized and analyzed using spline-fit functions. Upon examining both reconstructed fossil premaxillae and those of extant ruminants, Solounias et al. [41] and Solounias and Moelleken [42] found that snout shape correlates well with selectivity in extant species. Solounias et al. [41] also confirmed their inferences from snout shape using dental microwear data (see below).

Dompierre and Churcher [44] modified the method used by Solounias et al. [41] and Solounias and Moelleken [42] for their study of diet in extinct camelids. The original method, which relied on the curvature of snout profiles, necessitated scaling each profile to an equivalent size; the Premaxillary Shape Index (PSI) of Dompierre and Churcher [44] used area ratios and so removed the size component. PSI scores were found to correlate with diet in extant herbivores, such that the highest PSI scores were present in grazers and lower scores were present in selective browsers [44]. Dompierre and Churcher [44] concluded that PSI scores are potentially indicative of diet in extinct animals as well.

The relationship between snout shape and dietary habit has also been inferred outside of ungulate mammals. Christiansen [45] examined the relationship between muzzle breadth and body mass in sauropodomorph dinosaurs and found that breadth and mass were correlated, although there was no discussion of snout shape in relation to overall breadth. Additionally, snout breadth was measured at the premaxilla-maxilla suture, which occurs in markedly different places relative to the anterior-most point of the snout in macronarian and diplodocoid sauropods [34] and may have over-estimated snout breadth in narrow-snouted taxa as a result. Carrano et al. [46] examined the relationship between a suite of morphological characters (including snout breadth) and dietary preference in hadrosaur dinosaurs. The two subclades within Hadrosauridae, Lambeosaurinae and Hadrosaurinae, were found to be differentiated by relative snout breadth and limb proportions, similar to the pattern seen in ungulates. Carrano et al. [46] used this to infer an open-habitat, non-selective feeding behavior for the broad-snouted hadrosaurines and a closed-habitat, selective-feeding behavior for the narrow-snouted lambeosaurines. One hadrosaurine, *Prosaurolophus*, has a relative snout breadth within the range seen in the selectively browsing lambeosaurines [46]; this may suggest that there is not a perfect correlation between phylogeny and behavior. Similar, though less quantitative, inferences have also been made for ceratopsians [47] and thyreophorans [48], [49].

**Figure 1:**
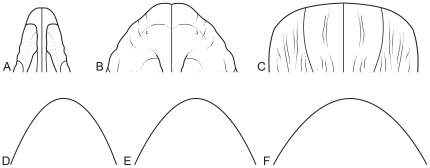
Snout shapes of sauropodomorph dinosaurs and ungulate mammals. Above: A) *Plateosaurus*, B) *Camarasaurus*, and C) *Diplodocus* snouts. Below: Outlines of snouts from a mammalian browser D), an intermediate feeder E), and a grazer F). Sauropod snouts modified from [154]; mammal snout outlines modified from [40].

Although sauropods were typically much larger than both large ungulates and large hadrosaurs, absolute skull size does not differ greatly between the two dinosaurian clades. Cranial volume estimates in sauropods range from ca. 0.05 m^3^ in *Dicraeosaurus* [50] to 0.2 m^3^ in *Brachiosaurus* [51], compared to to ca. 0.3 m^3^ in the hadrosaur *Edmontosaurus* [52]. As a result, snout breadths are roughly constrained within a similar morphospace (although some sauropod taxa greatly exceed the range of breadths seen in hadrosaurs, e.g., *Nigersaurus* [53]). Sauropod snouts also display a range of variation in shape, from narrow-snouted taxa like *Camarasaurus* to broader snouted taxa like *Diplodocus*, that is reminiscent of the pattern observed in modern ungulates (Fig. 1). It is reasonable to hypothesize that the snout morphologies noted in sauropods would have impacted selectivity and intake rate in much the same way that they do in extant ungulates, although in the absence of direct behavioral observations, additional evidence may needed. One source of such evidence is dental wear.

#### Dental microwear

**Figure 2:**
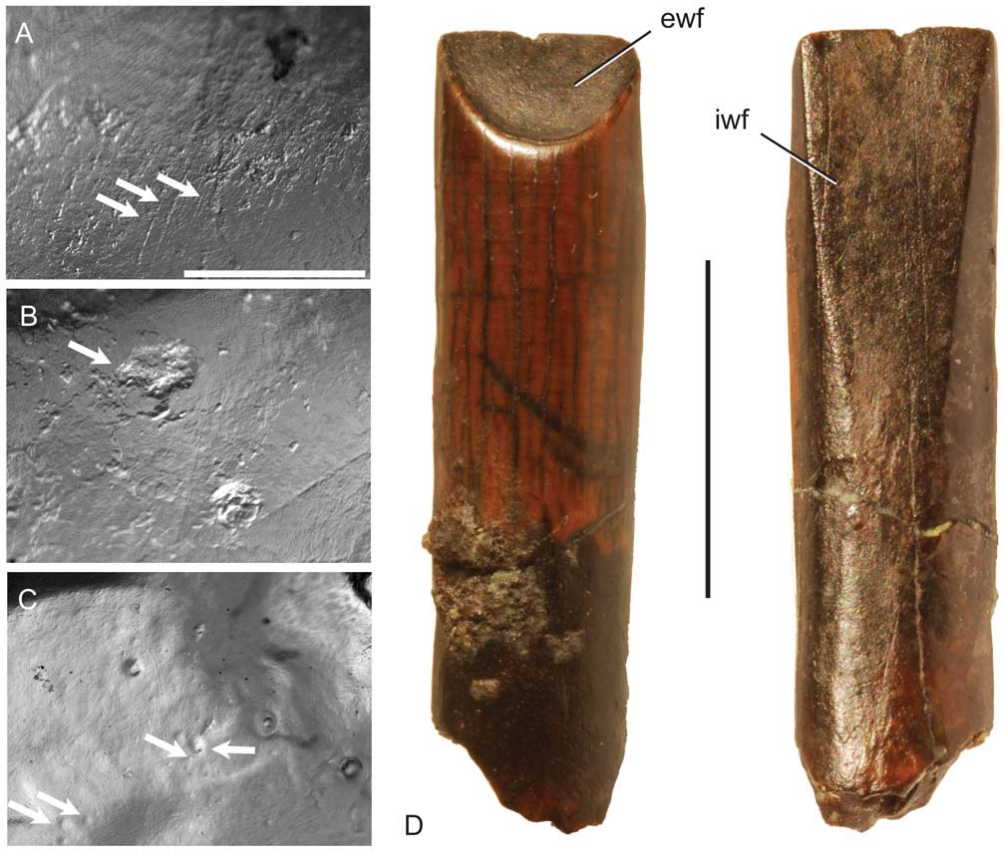
Examples of microwear features (exemplars indicated by arrows). A) Scratches, features at least 4× longer than wide. B) Gouges, large features with irregular margins. C) Pits, subcircular features, typically small. D) A tooth of *Nigersaurus*, illustrating the paired wear facets, labial (ewf) and lingual (lwf), seen on rebbachisaurid teeth. The labial facet is seen in most diplodocoid dentitions. Scale in A = 0.5 mm; B, C to scale with A. Scale in D = 1 cm. D is modified from [10].

Dental microwear is the study of damage done to teeth by contact with other surfaces, in particular the interactions between a tooth and food, grit, and opposing teeth. Studies of microwear typically include the quantification of features falling into one of the following three categories (Fig. 2; [54]):

**scratches**: features that are at least 4 times longer than wide
**pits**: deep, subcircular features
**gouges**: oblate features with irregular margins

Such features preserve information about the last few meals an organism ate and are one of the few direct lines of evidence we have for interpreting the diets of extinct organisms, particularly when those organisms have no extant descendants for comparison. Although caution must be used when interpreting diets of extinct organisms, broad dietary categories are often assignable. By providing direct evidence of what organisms ate, microwear features can also indicate overlap in resource exploitation and behavior.

The majority of microwear studies to date involve the reconstruction of diets in extinct and extant mammals [41], [54]–[60], based primarily on wear features recovered from molars and premolars. Increasingly, however, studies focusing on the dental microwear of non-mammalian organisms have begun to appear, including cynodonts [61], crurotarsans [61], dinosaurs [10], [22], [23], [30], [48], [62]–[64], and fish [65], [66]. Of these, only the analyses of microwear in stickleback fish [65], [66] were able to control diet experimentally using the organism itself; the rest relied upon comparisons with studies of mammalian microwear.

This reliance is a potential concern, due to broad differences in shape and function between the majority of teeth in question and the molariform teeth examined in the mammalian studies. Molars are used for oral processing of food in mammals, using a combination of puncturing, slicing, and crushing to break down foodstuffs. Non-mammalian animals typically have no oral processing (with the notable inferred exceptions of marginocephalian and ornithopod dinosaurs) and use their teeth solely for food-acquisition behaviors involving puncturing or slicing. Incisiform teeth, therefore, may prove a more useful analogue to archosaur teeth than molariform teeth.

The majority of incisor microwear studies have been done on primates, although isolated studies of non-primate incisor microwear exist [67]–[70]. There is evidence for a correlation between incisor wear and diet in primates [71]–[73] although some [74] suggest that incisor microwear is useful only for determining finer-scale dietary preference, once a broader category (e.g., frugivory) has been determined by other means.

Although the degree to which the manual manipulation of foodstuff by primates alters the character of wear is uncertain, some behaviors, such as leaf stripping, are strong candidates to broadly correspond with potential food acquisition behaviors in other organisms. Importantly, leaf stripping is known to leave characteristic microwear patterns on incisors in *Gorilla* [75]. There is also evidence that browse height impacts incisor microwear in predictable ways, with a greater proportion of large features appearing on the teeth of upper canopy feeders than on those of ground-level feeders as a result of the lower concentration of grit relative to phytoliths in food obtained at those greater heights [72], [76], [77]. In the Mesozoic, it is probable that phytoliths occurred in both understory plants (i.e., ferns; [78]) and upper-story conifers [79]. Studies of modern plants, however, suggest that ferns accumulate substantially less silica (primarily taken up as phytoliths) than other gymnosperms, such as conifers [79]; extrapolated to the Mesozoic, this suggests that an organism feeding on the ground cover would encounter a higher grit-to-phytolith ratio, as suggested above. Some understory plants (particularly *Equisetum*) are presumed to have been major silica accumulators, however [79], [80].

The only study to examine both molar and incisor microwear in an ungulate (*Equus quagga*, a grazer; [70]), found that patterns of molar microwear and incisor microwear were not similar, such that pits, rather than scratches, were the dominant feature on the incisors. This is potentially explained either as a function of exogenous grit, as the incisors would have been the first teeth to contact a foodstuff and would therefore encounter more of the grit, or as a function of selective use of incisors in feeding on more robust foodstuffs (e.g., woody plants) [70].

As noted above, several studies have discussed microwear features in sauropod dinosaurs, most commonly in diplodocoid sauropods [10], [22], [23], [30]. Although the features recovered from diplodocoid teeth (*Diplodocus* in [22], [23], [30]; *Nigersaurus* in [10]) are typically similar, primarily fine scratches, the interpretation of the behavior that caused these features differs, from high browsing (e.g., feeding on upper canopy vegetation) [22], [23] to branch stripping [30] to ground-level browsing [10]. The uncertainty over the functional significance of these wear features is likely exacerbated by two factors: first, sauropod microwear has previously been examined in the absence of other data, unlike what has been done for modern and extinct mammals; and second, sauropod diversity has been sampled only narrowly, and so there has been little relevant comparative data.

The influence of jaw motion on microwear features in sauropod dinosaurs is also worthy of discussion. The tooth row in diplodocoids is restricted anteriorly, resulting in a dentition that is primarily oriented transversely [34]. Because of this orientation, it is most probable that diplodocoid sauropods utilized a shearing bite with a primarily orthal motion, making their dentitions coarsely analogous to the incisors of many primates [71], [72], but not necessarily ungulates [70]. Although previous work [24], [26], [30] has suggested the possibility of propaliny in the bite stroke of diplodocids, the lack of marginal dentition in diplodocoids would mean that much of the fore-aft motion would be wasted during the slicing phase of the bite, as the transversely-oriented tooth row would only occlude for a fraction of the stroke before being taken out of alignment. Additionally, when propaliny has been proposed for other dinosaurs [64], [81], [82], it has been demonstrated as a means to prolong the occlusion and increase oral processing efficiency [83]. Sauropods, however, lack the fleshy cheek that enables oral processing in ornithischian herbivores [84], and the teeth were likely used purely for food acquisition rather than processing. It is most parsimonious to assume, therefore, that the functional component of the bite—where tooth met food—was orthal in nature. This does not mean that there could have been no palinal motion during the stroke; fore-aft movement of the lower jaw may have been used to accomplish occlusion of the upper and lower tooth rows prior to the final bite phase (as also happens in some mammals [85], [86]) or to widen the gape [26], [30]. It does suggest, however, that there was little to no retractive component of the bite stroke during a microwear-producing phase (i.e., where tooth-food contact occurred). Although the articulation in many diplodocoids is known only from the quadrate and not the lower jaw, most diplodocoid jaw joints are presumed to be similar [30], suggesting that any in-group variation in wear features perceived is most likely the result of diet and not jaw motion.

It is also important to note that much of the work on mammalian microwear surrounds the grazer-browser continuum, which necessarily cannot apply to Jurassic and Early Cretaceous dinosaurs due to the lack of grasses at that time [87]. Therefore, many of the assumptions about the relationship between microwear patterns and ground-height feeding behavior, such as the dominance of scratches over pits seen in grazers [56], must be re-examined. As seen elsewhere (fig. 8A in [56]), browsers and grazers separate along the scratch count axis, but browsers (including low, medium, and high browsers) separate primarily along the pit count axis (figs. 8A–D in [56]). It is probable, then, that the ratio of pits-to-scratches is more likely to be informative of diet in browsers such as sauropods.

Caution should be exercised when drawing parallels between microwear on mammalian teeth (including incisors) and that found on sauropod teeth. The chewing motion of an ungulate, with its transverse power stroke, is highly derived and without parallel in Sauropoda. Certain aspects of foodstuffs, particularly their toughness and grit content, however, can be expected to influence microwear features with reasonable consistency across taxa. Although the relationship between incisor microwear and diet is uncertain, inferences can still be drawn about the character of foodstuffs ingested. In particular, feature size and texture are expected to correspond with browse height and certain intrinsic plant properties, such as woody vs. herbaceous stems.

To develop a testable hypothesis for diplodocoid feeding behavior, evidence from snout shape and microwear will be compared, not just with data from modern herbivores, but also with each other. In this way, an approximation of the “total evidence” approach will be brought to bear on this question.

## Materials and Methods

Morphological data, in this case snout shape and microwear features, can be used to distinguish between several combinations of browse height (ground-height, mid-height, and upper canopy) and browse strategy (selective and nonselective) in diplodocoid sauropods. Here, ground-level feeding is defined as feeding on vegetation within 1 m of ground height, mid-height feeding is defined as feeding between ground height and 10 m, and upper canopy feeding is feeding at all heights above 10 m. Ten meters is chosen as the upper limit for mid-height browsing based on estimated maximum head height of diplodocoids in quadrupedal stance, using combined neck and forelimb height [30]. The following is summarized in Table 1.

**Table 1:** Feeding strategies and the predicted snout shape and microwear features associated with each.

Feeding strategy	Snout Shape	Microwear features
Browsing: Ground-height	Square	high proportion of pits relative to other features, fine scratches
Browsing: Mid-height (1–10 m)	Square/Round	fewer pits relative to other features
Browsing: Upper canopy	Square/Round	few pits
Browsing: Non-selective	Square	subparallel scratches, fine features (i.e. fine scratches, no pits)
Browsing: Selective	Round	cross-scratches, large features (i.e. coarse scratches, gouges)

### Evidence for browse height

Browse height was examined primarily through the examination of dental microwear features. The primary influence of browse height on microwear is through the creation of pits as a consequence of ingested grit. Grit, exogenous mineral particles, are suspended and transported in the air through aeolian processes and through the actions of animals (e.g., walking). These particles then fall out of suspension and are deposited on the ground or on plants. A proportionally larger amount of grit is deposited on plant surfaces at lower heights than those that greater heights [88], [89]. Taxa that browse at lower heights therefore typically ingest more extraneous grit, and their teeth may have a higher proportion of pits in their microwear features, as is seen in ground-height browsing ungulates [90]; this trend may be particularly exaggerated in the incisors of those animals [70]. Recent work also suggests that grit and dust does not have an additive effect on scratch counts in mammals [91], so we might expect to see the effect of increasing grit solely in the proportion of compressional features such as pits. Ground-height browsing sauropods will therefore be expected to have the highest proportion of pits in their microwear features, followed by mid-height and upper canopy browsers.

The incisors of ground-height feeding primates also display a slightly different character than those of taxa browsing at greater heights. Mean scratch breadth is lower in taxa browsing at or near ground height, as a function of mean particle size and the ratio of soil particles to phytoliths [72], [92]. Because the teeth of sauropod dinosaurs are assumed to be functionally analogous to the incisors of mammalian herbivores (see above), it is expected that the scratch breadth in ground-height browsing sauropods will also be smaller than in those that browse at different heights. Although a diet heavy on plants with substantial silica accumulation (e.g., modern *Equisetum*; [80]) may skew scratch breadth towards the broad end of the spectrum, such plants were rarely a dominant component of relevant ecosystems (see Environmental Signal, below), and silica accumulation in other ground-height forage is assumed to be lower than that of mid- and upper-canopy browse [79]. Additionally, the impact of woody browse (likely a component of selective browser diets) may overwhelm the signal from phytolith components, resulting in still broader scratches in selective browsers. Nonetheless, the potential impact of diet on scratch breadth will need to be accounted for where appropriate.

### Evidence for browsing strategy

Browsing strategy (selective vs. nonselective) was examined using both microwear and snout shape indices. Snout shapes in selective browsers were predicted to be narrower than those of nonselective browsers, as in herbivorous mammals. Two microwear features can indicate browse type: scratch orientation consistency and feature size. Consistency of scratch orientation has been related to food texture, such that softer (e.g., herbaceous) foods result in more unimodally distributed scratch orientations, whereas low consistency of orientation (i.e., cross-scratches) is related to eating harder or more brittle foods [93], [94]. Cross-scratching of this type has been seen on the shearing facets of cat carnassials, where it has been hypothesized to be the result of the multiple bite actions observed in those animals when attempting to bite through tough materials [93], [95]; movement of incompletely sheared foodstuffs across the facet during this process would create cross-scratching on a shearing facet [93], [96]. Note that this does not require complex jaw movements, as the cheek teeth in cats are in precise occlusion [93], [96]. Harder foods encountered by a sauropod would likely have included thick stems, indicating woody browse that is selectively browsed upon. Larger features (i.e., gouges, coarse scratches) also indicate selective browsing, either as a result of woody stems (coarse scratches) or large particles such as seeds or spores (gouges).

### Quantifying snout shape

Six genera of diplodocoid sauropod dinosaur (*Apatosaurus*, *Dicraeosaurus*, *Diplodocus*, *Nigersaurus*, *Suuwassea* and *Tornieria*) preserve enough of the skull to reconstruct the snout shape. The skulls of these six genera were reconstructed in dorsal view based on examination of the original materials (Table S1). These reconstructions are prone to some error, and the amount of material available necessarily influences their accuracy; *Diplodocus*, for example, is known from multiple articulated skulls, whereas *Dicraeosaurus* is known only from disarticulated braincases and fragmentary dermal elements. Nonetheless, these were produced with as much rigor as possible, and so should vary in ways consistent with their variation in life.

**Figure 3:**
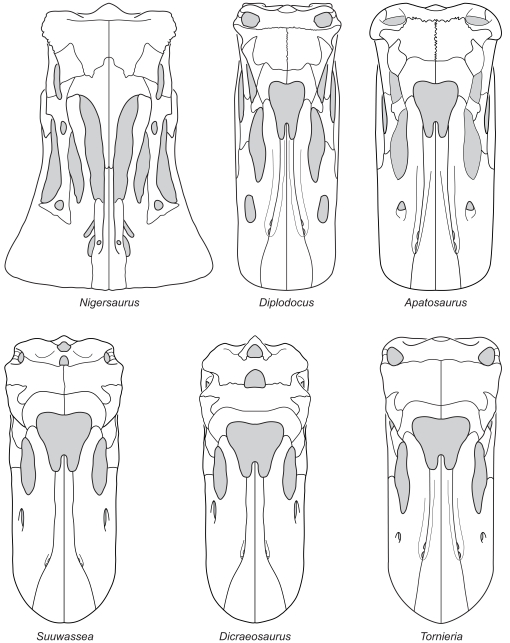
Reconstructions of diplodocoid skulls used in this analysis. Reconstructions of *Nigersaurus* and *Diplodocus* modified from [10] and [34], respectively. All other reconstructions based on material listed in Table S1. Skulls scaled to equivalent anteroposterior lengths.

These reconstructions (Fig. 3) were measured using two squareness indices: the upper arcade index (uAI) and the premaxillary-maxillary index (PMI). A third method, measuring the divergence angle of the premaxillae, enables the direct measurement of squareness from fossils, as it does not require a reconstruction to remove deformation. These indices were then compared among taxa, first to test the null hypothesis that there is no difference in snout squareness among diplodocoids, and second to test the hypothesis that any variation in snout shape is correlated with phylogeny, not diet. The non-diplodocoid neosauropods *Brachiosaurus* and *Camarasaurus* were also examined, as taxa widely recognized as relatively selective browsers and as outgroups to the diplodocoids. To further elucidate trends in snout shape, the basal eusauropods *Jobaria* (based on [97]), *Mamenchisaurus* (based on [98]; this shape is extrapolated from a dentary), *Patagosaurus* (extrapolated from the dentary MPEF-PV 1670), and *Shunosaurus* (extrapolated from the dentary ZG65430) were also examined, as was the basal sauropodomorph *Plateosaurus* (based on [34]). In the absence of microwear or other contextual data for these latter organisms, interpretation of their feeding behavior is limited.

#### uAI

**Figure 4:**
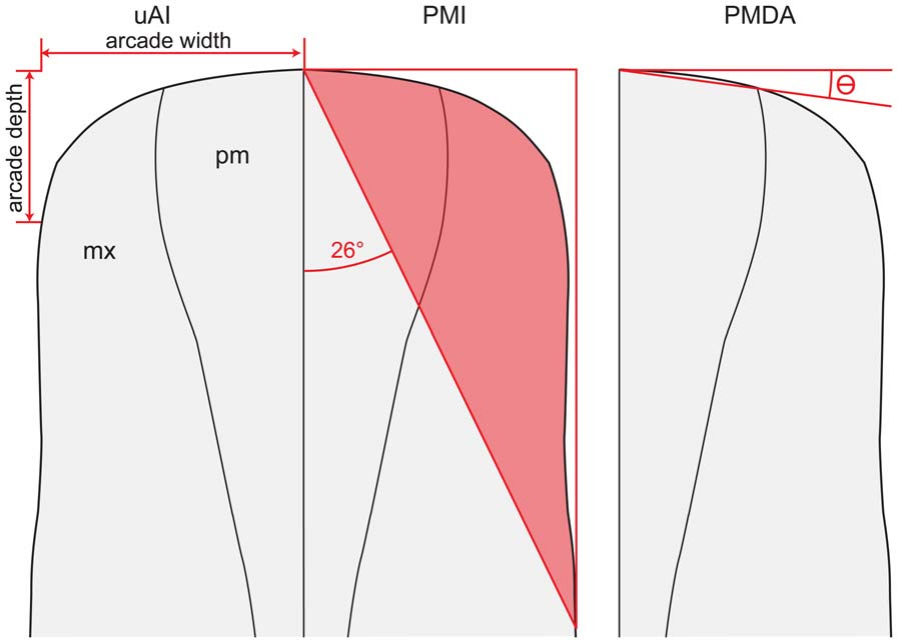
Metrics used to determine snout shape in this study. Snout depicted based on *Diplodocus* in Figure 3, anterior towards top of page. From left to right: the upper arcade index (uAI) measures snout breadth by taking the ratio of arcade width to arcade depth (higher numbers indicate squarer snouts); the premaxilla-maxilla index (PMI) is determined by taking the ratio of an area covered by the snout within a predetermined triangle to the area of that triangle (higher numbers indicate squarer snouts); the premaxillary divergence angle (PMDA) determines squareness by measuring the divergence of the anterior margin of the premaxilla from perfectly square (higher numbers indicate greater divergence from square and therefore roundness). The angle of the hypotenuse of the triangle used to calculate PMI (26°; 64° internal angle) is based on the angle used in the PMI's parent metric, the PSI [44].

The uAI is a modification of the AI introduced by Boué [34]. Although the original metric measured the ratio of the depth and breadth in the lower dental arcade, the uAI utilizes the upper jaw instead, because upper jaws are more commonly preserved in diplodocoid dinosaurs. In order to retain 1.0 as the dividing score between square and round jaws, the uAI measures only the right or left half of the snout (Fig. 4), although Boué's metric included both right and left jaws in its width measurement. In both metrics, the measured variable is the width of the dental arcade (in the uAI, the half-width) divided by the anteroposterior depth.

#### PMI

The PMI is also a modification of an older metric; in this case it is a modification of the premaxillary shape index (PSI) [44]. Because the sauropod snout includes both the premaxilla and the maxilla, the PSI is only slightly modified as the premaxillary-maxillary index (PMI). This index is otherwise calculated in much the same way as the PSI (Fig. 4). First, a line is drawn perpendicular to the sagittal midline of the skull and tangent to the anterior-most point on the skull. A second line is drawn parallel to the midline tangent to the broadest point of the snout. A third line is then drawn at 26° from the long axis of the skull connecting the first two lines, forming a right triangle; 26° is used to be consistent both with the PSI and with work predating the PSI. The area of the skull within that triangle is calculated (using the measure tool in Adobe Photoshop CS4) and compared to the area of the triangle as a whole to compute the PMI. In cases where the snout narrows behind its broadest part (e.g., *Nigersaurus*, certain hadrosaurs), the shape posterior to the broadest point is disregarded, and the snout is considered to have continued in a straight line to the point of intersection with the hypotenuse. Although this may inflate the PMI score slightly, it more accurately reflects the effective shape of the snout.

#### Premaxilla Divergence Angle

Due to the often fragmentary or deformed nature of fossil material, the prior two metrics rely on reconstructions of skulls; measurements based on deformed materials would necessarily represent an unrealistic shape. Although every effort was made to accurately reconstruct morphology, this has resulted in a small sample size. One method to measure squareness directly from fossil material (reducing potential error inherent in reconstruction) and increase sample size involves comparing the angles of divergence on the anterior margin of the premaxilla (PMDA). This is measured here by orienting the specimen in strict dorsal or ventral view and measuring the angle formed between the anterolateral and anteromedial corners of the premaxilla and a line drawn perpendicular to symphysis (Fig. 4).

### Quantifying microwear

Forty-seven teeth belonging to seven genera were examined for microwear features (Table S2); of these, only 12 were subsequently deemed suitable for analysis (Table 4). Damaged or heavily weathered teeth were deemed unsuitable and not examined, following [99]. The suitable teeth were molded using a high-resolution polyvinylsiloxane dental molding material (Coltene Whaledent President microSystem 6012). Casts were made in water-clear epoxy and examined at 70× using transmitted light microscopy, following [56]. Images were taken using a Spot CCD camera (Spot Insight 11.2 Color Mosaic, Diagnostic Instruments) at the highest resolution available (36 bits/pixel, 300 dpi), mounted on a Nikon SMZ 1500 microscope. Image analysis was performed in Microware 4.02 [100]. Where possible, wear was examined from multiple sites on the same tooth and averaged. Features measured were scratch number, scratch orientation, pit number, and pit size. Patterns of microwear were examined against patterns characteristic of feeding behavior in modern mammals (see below). Following recent criticisms of purely quantitative analyses of microwear [101], analysis of microwear features is based solely on qualitative comparisons (e.g., average size, shape) and feature-to-feature ratios; raw counts and counts per unit area are provided but not analyzed.

Although sauropod remains are abundant globally, skulls are a rare component of sauropod fossil assemblages [32]. Even Diplodocoidea, the clade most completely represented by cranial material, contains only six genera with tooth-bearing elements associated with them (see above). Isolated, shed teeth are common finds, but are not identifiable to a useful degree, and can only be assigned to large clades (e.g., Diplodocoidea). In some instances, particularly involving Morrison Formation sauropods, isolated dentigerous elements are also not identifiable to the genus level, due to the paucity of non-basicranial autapomorphies in the clade. Even among those teeth that are both worn and assignable to a particular taxon, preserved wear features are rare, resulting in the relatively small sample size seen here.

### Statistical analyses

The distributions of three variables were examined statistically: snout shape (for browsing strategy), scratch breadth (for browse height), and pit/gouge size (for both height and strategy). For two-sample comparisons, non-parametric Mann-Whitney U tests were performed; sample sizes for snout shape were too small to meet normality criteria, and both scratch breadth and pit/gouge size failed a normality test (Shapiro-Wilk) for all samples. The Kruskal-Wallis test (an extension of the Mann-Whitney U) was used for instances where multiple comparisons were desirable. For significant results of Kruskal-Wallis tests, pairwise comparisons (following the method of [102]) with an adjusted P-value were used to determine which samples were distinguishable from each other.

### Institutional Abbreviations

**AMNH**, American Museum of Natural History, New York, New York, USA; **ANSP**, Academy of Natural Sciences, Philadelphia, Pennsylvania, USA; **CM**, Carnegie Museum of Natural History, Pittsburgh, Pennsylvania, USA; **CMC**, Cincinnati Museum Center, Cincinnati, Ohio, USA; **CMN**, Canadian Museum of Nature, Ottawa, Ontario, Canada; **CPC**, Colección Paleontológica de

Coahuila, Saltillo, Coahuila, Mexico; **MB.R.**, Humboldt Museum für Naturkunde, Berlin, Germany; **MNN**, Museé National du Niger, Niamey, Niger; **MPEF**, Museo Paleontológico Egidio Feruglio, Trelew, Chubut, Argentina; **MOR**, Museum of the Rockies, Bozeman, Montana, USA; **PU**, Museum of Natural History, Princeton University, Princeton, New Jersey, USA; **ROM**, Royal Ontario Museum, Toronto, Ontario, Canada; **RTMP**, Royal Tyrell Museum of Palaeontology, Drumheller, Alberta, Canada; **USNM**, United States National Museum, Washington, D.C., USA; **UUVP**, University of Utah, Salt Lake City, Utah, USA; **YPM**, Yale Peabody Museum, New Haven, Connecticut, USA; **ZG**, Zigong Dinosaur Museum, Zigong, Sichuan, People's Republic of China.

## Results

### Snout shape

Results of the snout shape analyses (uAI, PMI, PMDA) are summarized in Tables 2 and 3.

**Table 2:** Snout shape scores by taxon.

	uAI	PMI	PMDA
*Apatosaurus*	1.5	84%	6.3°
*Dicraeosaurus*	0.6	74%	24.4°
*Diplodocus*	1.2	84%	7.4°
*Nigersaurus*	4.0	95%	3.5°
*Suuwassea*	—	74%	25°
*Tornieria*	—	71%	25.4°
*Brachiosaurus*	0.6	68%	33°
*Camarasaurus*	0.4	63%	40°

**Table 3:** PMDA scores by element.

Containing clade	Family	Genus	Species	Specimen #	PMDA	Position
Diplodocoidea	Diplodocidae	*Diplodocus*	*longus*	AMNH 969	4.5	L
					7	R
				USNM 2673	9.8	L
			*carnegii*	CMNH 11161	8.3	L
		*Apatosaurus*	*sp.*	CMNH 11162	12.4	L
					4	R
			*sp.*	CMC VP 7800	3	L
					5.8	R
		*Tornieria*	*africana*	MB.R.2346	25.4	L
	Dicraeosauridae	*Suuwassea*	*emilieae*	ANS 21122	25	L
		*Dicraeosaurus*	*sp.*	MB.R.2339	24.4	L
	Rebbachisauridae	*Nigersaurus*	*taqueti*	MNN GAD-512	3.5	L
Macronaria		*Brachiosaurus*	*brancai*		34.5	L
					30.2	R
		*Camarasaurus*	*lentus*	CM 11338	45.4	R
					46.5	L
			*sp.*	UUVP 3999	27	R

#### uAI

Four taxa (*Apatosaurus*, *Dicraeosaurus*, *Diplodocus*, and *Nigersaurus*) preserve enough of the maxilla that the uAI can be calculated. This metric could not be determined for *Suuwassea* and *Tornieria*; the only known maxillae of those taxa are distally incomplete and the position of the posterior-most tooth cannot be accurately determined. *Nigersaurus* had the highest uAI score and therefore the squarest snout (4.0), followed by *Apatosaurus* (1.5) and *Diplodocus* (1.2). *Dicraeosaurus* has the lowest uAI score (0.6). The non-diplodocoid sauropods *Brachiosaurus* and *Camarasaurus* have uAI scores similar to those of *Dicraeosaurus* (0.6 and 0.4, respectively).

#### PMI

As above, *Nigersaurus* had the highest PMI value (95%). *Apatosaurus* (84%) and *Diplodocus* (84%) had the next highest PMI, followed by *Dicraeosaurus* (74%) and *Suuwassea* (74%). *Tornieria* (71%) had the lowest PMI score of the ingroup taxa. Diplodocoids had higher PMI scores (and therefore squarer snouts) than all outgroup taxa (*Brachiosaurus* = 68%; *Camarasaurus* = 63%). Snouts of the basal eusaruopods *Jobaria* (55%), *Mamenchisaurus* (58%), and *Shunosaurus* (58%) were rounder than those of all examined neosauropods, although *Patagosaurus* (65%) has a snout shape intermediate between *Brachiosaurus* and *Camarasaurus*. The basal sauropodomorph *Plateosaurus* (44%) was roundest of all.

#### Premaxillary Divergence Angle

The initial sample included 16 premaxillae. Of those 16, 12 were deemed complete enough to measure (Table 3). In the incomplete premaxillae, the thin ‘lateral plate’ [30] that extends ventrally from the labial margin of the premaxilla and forms the ventral-most portion of the element was missing or heavily damaged, preventing accurate assessment of the PMDA from those elements.

The diplodocoids again appear to have segregated into two groups, with *Dicraeosaurus* (24°), *Suuwassea* (25°), and *Tornieria* (25°) having high divergence angles compared to *Apatosaurus* (6°), *Diplodocus* (7°), and *Nigersaurus* (4°). The snout of *Nigersaurus* is again the squarest; those of *Suuwassea* and *Tornieria* are the roundest. The difference between *Nigersaurus* and *Apatosaurus* is less marked (2°) than with previous metrics. The PMDA of *Brachiosaurus* (35°, 30°; average = 33°) and *Camarasaurus* (27°, 45.4°, 46.5°; average = 40°) are higher than for any diplodocoid. The variation observed in *Camarasaurus* is potentially the result of ontogeny; the element that provided the 27° PMDA measurement (UUVP 3999) is much larger and presumably came from an adult, whereas CM 11338 is a subadult individual.

### Microwear

**Figure 5:**
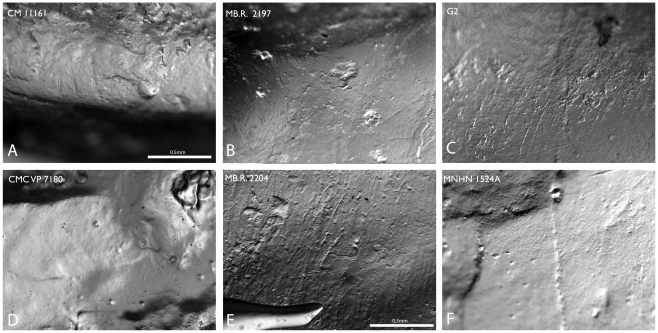
Microwear features recovered from sauropod dinosaurs. A, *Diplodocus*. B, E, *Dicraeosaurus*. C, *Nigersaurus*. D, *Apatosaurus*. F. c.f. *Rebbachisaurus*. A, C, D, and F dominated by small pits and fine scratches, interpreted as indications of ground-height, non-selective browsing; B and E are dominated by large gouges and coarse scratches, interpreted as indications of mid-height, selective browsing. A–F to scale; scale bar in A, E = 0.5 mm.

Although 47 teeth were examined for microwear features, only 11 (23%) were found to have features that record diet. Of these, only seven teeth belonging to five taxa (*Brachiosaurus*, *Camarasaurus*, *Dicraeosaurus*, *Diplodocus*, and *Nigersaurus*) appeared to preserve an accurate sample of wear. Figure 5 illustrates representative wear features for these five taxa. The remaining four teeth preserve microwear that cannot be directly attributed to diet, either due to taphonomic alteration (*Apatosaurus*), location (not on the wear facet itself; *Diplodocus* USNM 2673, *Nigersaurus* G100), or because the preserved features are so few in number (*Rebbachisaurus*).These final four teeth and their microwear features are described below and compared qualitatively. Full results are presented in Table 4; raw data (including individual feature dimensions) are available in Table S3.

**Table 4:** Microwear features recovered from teeth.

Taxon	Specimen	Pit #	L∶B	Area (µm^2^)	Circ. (µm)	S. #	S. L. (µm)	S. B. (µm)	C.S.
*Apatosaurus*	CMC VP7180	109	1.27∶1	73.37	46.43	—	—	—	n/a
*Dicraeosaurus*	MB.R. 2204D	359	1.64∶1	99.74	31.32	228	54.93	3.73	Y
	MB.R. 2204E	227	1:75∶1	160.06	43.75	176	49.12	4.93	Y
*Diplodocus*	CM 11161 LP1	14	1.28∶1	33.43	24.90	19	94.35	2.65	N
	CM 11161 RP2	37	1.38∶1	16.54	18.43	26	52.46	3.4	N
	USNM 2673*	15	1.32∶1	423.26	69.17	37	236.17	4.33	Y
*Nigersaurus*	G2	276	1.48∶1	34.64	26.80	138	678.49	2.69	N
	G100*	9	2.28∶1	208.04	70.19	30	137.53	3.84	Y
*Rebbachisaurus*	MNHN 1512a	14	1.2∶1	174.73	69.17	7	166.29	3.57	Y
*Brachiosaurus*	MB.R. 2190	49	1.48∶1	520.57	51.12	72	57.52	3.86	Y
*Camarasaurus*	UUVP 1949	124	1.95∶1	80.03	31.83	99	47.32	3.76	N
	UUVP 3986	203	1.74∶1	93.46	33.31	185	80.21	4.02	Y

Abbreviations: L∶B, average length∶breadth ratio of pits/gouges; S. #, number of scratches; S. L., average scratch length; S. B., average scratch breadth; C.S., cross-scratches.

#### Apatosaurus

The probable *Apatosaurus* skull CMC VP 7180 contains multiple teeth in-situ, although the majority of these teeth were broken apically. Wear features were only recovered from one tooth, the first tooth in the left dentary. Although this tooth was heavily abraded post-mortem, as indicated by a ‘sugary’ texture [54], many small, subcircular features can be seen dotting the surface of the dentine. No scratches are recorded in the dentin, and no features were recovered from the enamel of any tooth.

#### Dicraeosaurus

Two teeth (MB.R. 2204 and 2197), tentatively assigned to *D. hansemanni*, preserve some microwear features. Features are recovered from both dentine and enamel surfaces of MB.R. 2204. On both surfaces, pits and gouges outnumber scratches, and large irregular features (gouges) are more common than the smaller, subcircular pits. On both surfaces, scratches lack a preferred orientation and cross-scratches are common. Features on the dentine are finer than those on the enamel surface.

The second tooth, MB.R. 2197, does not preserve a large amount of microwear, although some features are present on the labial enamel edge. Here, two exceptionally large gouges are preserved. No scratches or smaller pits were observed, and it is probable (although not certain) that these gouges are not the result of tooth-food or tooth-tooth contact.

#### Diplodocus

Three teeth from two specimens (CM 11161 and USNM 2673) preserve microwear features on the enamel.

Microwear features were recovered from small areas of the labial enamel margin of the facet on the second right premaxillary tooth and the first left premaxillary tooth of CM 11161. Identification of features was hampered here and on other diplodocid specimens by the application of a preservative lacquer, likely around the turn of the century. Attempts to remove this lacquer using alcohol-based solvents were generally unsuccessful.

As also seen in *Dicraeosaurus*, pits and gouges outnumber scratches on the enamel of teeth from CM 11161. Of the two features, small subcircular pits are substantially more common than large gouges. Scratches are generally subparallel, although cross-scratching does rarely occur, and oriented roughly along the apicobasal axis. Scratches extend over the edge of the facet and onto the lingual surface of the tooth for a short distance.

Seven loose teeth are associated with the *Diplodocus longus* skull USNM 2673. None of these teeth have wear facets, and based on the size and position of one tooth, some of them may have been unworn replacement teeth. One tooth, however, an incomplete crown recovered separately from the other six loose teeth, does preserve what appear to be wear features on the presumed labial surface some distance from the apex. Based on size comparisons with the intact teeth of USNM 2673, this crown appears to be an upper tooth.

The wear features recovered from this surface differ from those seen on the labial facet in CM 11161 in both size and character. Scratches are substantially longer and broader than those from the facet margin. They are generally sub-parallel with rare-cross scratching, but the orientation is nearly perpendicular to the long axis of the tooth, with only a slight apicobasal component. Pits are also larger than those observed on the facet, although they are proportionally identical to those from CM 11161. Because these features do not come from either a facet or from the apical surface of the crown, they cannot be confirmed as the result of a bite stroke.

#### Nigersaurus

Wear features were recovered from two crowns, one each from the the G2 and G100 assemblages.

Wear features were observed on the labial enamel margin of the labial facet on the G2 crown. As in *Diplodocus*, scratches are elongate and narrow. Scratch orientation is generally apicobasal, without cross-scratching. Small pits are the most common feature on the facet. The proportions of these pits are more oblate than those of *Diplodocus* and *Apatosaurus*, but substantially rounder than those observed in the enamel of *Dicraeosaurus*.

No wear features were recovered from the facet of the crown from G100, perhaps as a result of post-mortem wear. Features were observed on the lingual surface of the enamel basal to the actively worn surface. Of the features observed, the majority were scratches. All features were generally larger than those observed on the facet of G2. Similar to the wear recovered from USNM 2673, scratch orientation is more mesiodistal than apicobasal, although there is still a minor apicobasal component. Cross scratching occurs but is rare. Pit size was also substantially larger than those seen in the G2 tooth. Because these features are located away from surfaces of active wear, it is likely that they were caused by some contact outside of the bite stroke.

#### Rebbachisaurus

A single loose tooth of rebbachisaurid type (MNHN 1512a; see [103]) is referable to *Rebbachisaurus*. Wear features were recovered from the enamel near the smaller facet (presumably the labial surface). Scratches were very long and narrow. Cross scratching did occur, although scratches appear to be predominantly mesiodistally oriented. Pits were quite large but generally subcircular.

#### Camarasaurus

Two teeth (UUVP 1949, UUVP 3986) preserved a substantial amount of quantifiable microwear features. In both cases, pits outnumber scratches. The pits are large, similar in size to those seen in *Dicraeosaurus*. Scratch length varies but is generally shorter than that seen in *Diplodocus* and *Nigersaurus*. Scratch breadth is broad and most similar to that seen in *Dicraeosaurus*. Cross scratches are rarer than in *Dicraeosaurus* but do occur.

#### Brachiosaurus

Wear features were recovered from a single tooth, MB.R. 2190. Scratches dominate the preserved features. The pits that are preserved are often quite large, larger than but most similar to those preserved in *Dicraeosaurus*. These largest pits are typical of the wear found at the outermost margin of the facet; basal to the facet, the pits are more typical of the size seen in *Diplodocus* and *Nigersaurus*. Scratches are comparatively short, and cross-scratches are common. Scratch breadth is intermediate, occurring between the ranges of *Diplodocus* and *Dicraeosaurus*.

## Discussion

### Snout Shape

The six ingroup taxa examined can be broadly divided into two categories: square and round. Square-snouted diplodocoids include the diplodocids *Apatosaurus* and *Diplodocus* and the rebbachisaurid *Nigersaurus*. Round-snouted diplodocoids include the dicraeosaurids *Dicraeosaurus* and *Suuwassea*, and the diplodocid *Tornieria*. Round-snouted diplodocoids are generally squarer in profile than outgroup taxa such as *Brachiosaurus* and *Camarasaurus*, however.

Testing this statistically proves somewhat problematic, however. Grouping the sauropods a priori into three groups—“square” diplodocoids (*Apatosaurus*, *Diplodocus*, and *Nigersaurus*), “round” diplodocoids (*Dicraeosaurus*, *Suuwassea*, and *Tornieria*), and outgroup sauropods (*Brachiosaurus* and *Camarasaurus*)—permits examination of any differences in their means, although at such low sample sizes, the power of statistical tests to avoid Type II errors (erroneously failing to reject the null hypothesis) is reduced, and two populations may be construed as falsely similar. A Kruskal-Wallis test rejects the null hypothesis, that all samples have the same median, for both PMI (H = 6.402, d.f. = 2, P = 0.041) and PMDA scores (H = 13.176, d.f. = 2, P = 0.001). Pairwise comparisons found that, for both metrics, only the square population could be distinguished from the outgroup (P = 0.038). All other pairwise comparisons (round vs. outgroup, P = 0.773; square vs. round, P = 0.387) were found to be indistinguishable. The small sample size hampers the power of statistical tests to separate these groups, however.

This pattern of snout shapes is reminiscent of the pattern seen in modern ungulates. In light of that comparison, the broad-snoutedness seen in *Apatosaurus*, *Diplodocus*, and *Nigersaurus* is interpreted as an adaptation to ground-level, non-selective browsing in at least some diplodocoid sauropods. The narrower, rounded snouts of *Dicraeosaurus*, *Suuwassea*, and *Tornieria* are interpreted as evidence for greater subsistence on mid-height, selective browse, although the intermediacy of these shapes between the squarest diplodocoids and the roundest outgroups may indicate at least partial reliance on non-selective browsing in these taxa.

The snout shapes of macronarian sauropods like *Brachiosaurus* and *Camarasaurus* are consistently rounder than those of diplodocoid sauropods. If this difference is reflective of a different feeding behavior, it may be due to the difference in browse height. *Brachiosaurus* and *Camarasaurus* were both probably mid- to upper-canopy browsers, based on studies of neck posture [27], [28], [104], [105] (but see [106]) and limb proportions [30], [34], [107]. Diplodocids and dicraeosaurids, however, were likely browsing near or at ground level [27], [28], [30]. Differences in available vegetation at such lower heights (herbaceous plants at low heights, woody browse at mid- and upper heights), potentially resulting in the occasional non-selective exploitation of browse, may have influenced the relative breadth of the snout in *Dicraeosaurus*, *Suuwassea*, and *Tornieria*. Alternatively, there may be a phylogenetic component to the difference in snout shapes between selectively browsing diplodocoids and macronarians—if broad snouts are plesiomorphic for Diplodocoidea, there may be some constraint on the degree of roundness attained.

Examination of sauropod taxa outside the neosauropod radiation (*Jobaria*, *Mamenchisaurus*, *Patagosaurus*, and *Shunosaurus*) suggests that neosauropods as a whole display increased snout squareness over more basal taxa (U = 1, P = 0.01), although taxa like *Patagosaurus* appear to have achieved macronarian-type snout broadness early in eusauropod evolution. Eusauropods, in turn, are squarer than basal sauropodomorphs like *Plateosaurus*. This general trend towards increasingly square snouts may be related to an increased reliance on bulk herbivory [30], [108] and is possibly also related to the observed trend towards larger tooth crowns in sauropod and eusauropod dinosaurs [9]. The variation seen in basal eusauropods may be related to feeding behavior, but more research (particularly the acquisition of microwear data) is required to fully evaluate this hypothesis.

#### Comparisons with hadrosaurids

As discussed previously (see *Reconstructing Diets*, above), Carrano et al. [46] noted a general dichotomy of form between hadrosaurine and lambeosaurine hadrosaurs—hadrosaurines had broad snouts, lambeosaurines had narrow snouts—and related this difference to a division in dietary habit, such that the hadrosaurines (H) were interpreted as non-selective browsers and the lambeosaurines (L) were selective browsers. As large bodied dinosaurian herbivores, hadrosaurids are a potentially useful comparison for sauropods, particularly with regard to snout shape. PMI scores were determined for *Anatotitan copei* (H; [109]; this may be referrable to *Edmontosaurus*, however [110]), *Edmontosaurus regalis* (H; [109]), *Maiasaura peeblesorum* (H; [111]), *Prosaurolophus maximus* (H; [112]), *Saurolophus osborni* (H; [113]), *Corythosaurus sp.* (L; CMN 34825), *Hypacrosaurus altispinus* (L; ROM 702), *Lambeosaurus sp.* (L; ROM 758) and *Velafrons coahuilensis* (L; [114]). Although the premaxilla would have been covered in a keratinous beak in vivo, it is not unreasonable to assume the shape in life closely mirrored that of the bony supporting elements beneath. Although CMN 34825 and ROM 758 most likely represent juveniles, no study has yet noted shape change associated with the snout through ontogeny in ornithopods [115]–[117] or other dinosaurs, excluding only *Diplodocus* [32]. Furthermore, examination of an embryonic specimen of *Hypacrosaurus* (RTMP 87.79.334, [115]) results in an identical PMI to that of the adult (ROM 702), suggesting that snout shape was conserved in these taxa. Snout shape (PMI) scores and material used for reconstructions are given in Table S4.

**Figure 6:**
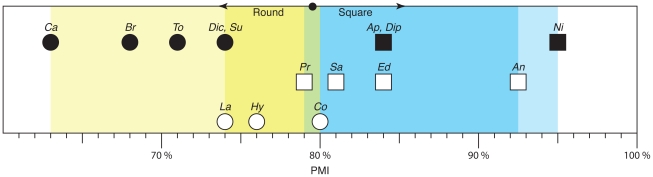
Plot of PMI scores for hadrosaurid (open symbols) and sauropod (closed symbols) dinosaurs. The vertical axis separates taxa into sauropods (top), hadrosaurines (middle) and lambeosaurines (bottom). Squares represent taxa considered to have been non-selective browsers, circles represent taxa considered to have been selective browsers. Blue tones indicate the range of square snouts and yellow tones indicate the range of round snouts; green tone indicates overlap; dark blue/dark yellow represent the limits of hadrosaurian snout shape diversity. Overlap in snout shape occurs between behavioral guilds in hadrosaurs, but not in sauropods, although sample size is limited for sauropods. Sauropod snout shapes are also more disparate than snout shapes in hadrosaurids. Inferences of hadrosaur diet based on [46]. Abbreviations: *An*, *Anatotitan*; *Ap*, *Apatosaurus*; *Br*, *Brachiosaurus*; *Ca Camarasaurus*; *Co*, *Corythosaurus*; *Dic*, *Dicraeosaurus*; *Dip*, *Diplodocus*; *Ed*, *Edmontosaurus*, *Hy*, *Hypacrosaurus*; *La*, *Lambeosaurus*; *Ni*, *Nigersaurus*; *Pr*, *Prosaurolophus*; *Sa*, *Saurolophus*; *Su*, *Suuwassea*; *To*, *Tornieria*.

Broad-snouted diplodocids group within the range of PMI scores seen in hadrosaurines; in both clades, scores cluster around 80–85% (Fig. 6; only those taxa examined by Carrano et al. [46] are plotted). The exceedingly square snout of *Nigersaurus* (95%) is nearly matched by that of *Anatotitan* (93%). Lambeosaurines, particularly *Hypacrosaurus* (76%) and *Lambeosaurus* (74%), typically have rounder snouts than hadrosaurines, although there is some overlap (e.g., *Corythosaurus*, 80%). There is also overlap in the range of scores between the upper end of the round-snouted diplodocoid range (*Dicraeosaurus*, 74%) and lower end of the lambeosaurine range (*Lambeosaurus*, 74%), although the PMI of *Tornieria* (71%) is lower than in any hadrosaurid examined. The lambeosaurine *Velafrons*, which was not included by Carrano et al. [46] and was not plotted in Figure 6, has a PMI score (85%) more similar to those of square-snouted diplodocids and hadrosaurines, which suggests the potential for non-selective browsing in Lambeosaurinae.

A Mann-Whitney U test comparing the PMI scores recorded for combined diplodocoid and hadrosaurid “square” (*Apatosaurus*, *Diplodocus*, *Nigersaurus*, and the hadrosaurines) and “round” (*Dicraeosaurus*, *Suuwassea*, *Tornieria*, and the lambeosaurines) groups demonstrates that the two groups can be distinguished (U = 1, P = 0.002). The overall similarity between snout shape in putatively nonselective and selective browsers in both diplodocoids and hadrosaurids suggests that a) PMI is a valid measure of an ecomorphological variable and b) hypotheses of non-selective and selective browsing based on snout shape in diplodocoid sauropods are well-founded.

### Microwear

Although the relationship between wear on molar and incisiform teeth is uncertain, the pattern of variation in wear features between round-snouted (*Dicraeosaurus*) and square-snouted (*Apatosaurus*, *Diplodocus*, *Nigersaurus*) diplodocoids is quite similar to that expressed in the molariform teeth of mammals. *Dicraeosaurus* featured larger, coarser features in general, with less orientational consistency than in the square-snouted taxa. These features can be tentatively interpreted as evidence for variation in both browse height and type (e.g., woody vs. herbaceous, high vs. low concentrations of phytoliths/sclerenchyma, selective vs. non-selective).

#### Browse height

*Dicraeosaurus*, *Diplodocus*, and *Nigersaurus* all had a large proportion of pits in their microwear features. *Nigersaurus* had the highest proportion of pits to scratches (2∶1), followed by *Dicraeosaurus* (1.3∶1) and *Diplodocus* (1.1∶1); the lowest proportion occurred in the putatively higher-browsing taxon *Brachiosaurus* (0.7∶1), although the mid-height browser *Camarasaurus* had a ratio similar to that of *Diplodocus* and *Dicraeosaurus* (1.2∶1). The ratio of pits to scratches in *Diplodocus* may be artificially low; only 96 total features were recovered from two teeth (compared to 414 in *Nigersaurus* and 403 in *Dicraeosaurus*). The proportion of those features varies between teeth, such that the first left premaxillary tooth has a pit∶scratch ratio of 0.7∶1, while the second right premaxillary tooth has a ratio of 1.4∶1. Regardless, it is clear that *Nigersaurus* has a higher proportion of pits to scratches than *Dicraeosaurus*, which suggests that *Nigersaurus* ate at a lower browse height (i.e., at ground level). The same is potentially true for *Diplodocus*, even allowing for the potentially misleading results from the left premaxillary tooth. The similarity in pits-to-scratches ratios in *Diplodocus*, *Camarasaurus* and *Dicraeosaurus* may indicate some overlap in browse height (and indeed, the two categories do overlap as defined here; Table 1), although the distinctly larger features present in *Camarasaurus* and *Dicraeosaurus* (see below) suggest that the subcircular features in each taxon had different root causes, most likely different browse types.

#### Browse type

Square-snouted diplodocoids *Diplodocus* and *Nigersaurus* are characterized by an abundance of fine (breadth <3.5 µm), subparallel scratches; round-snouted taxa such as *Camarasaurus* and *Dicraeosaurus* are dominated by coarse (breadth >3.75 µm) scratches, including a large proportion of cross-scratches. Following a significant Kruskal-Wallace result (H = 179.34, d.f. = 4, P = 0.000), pairwise comparisons found that the pairs *Diplodocus* and *Nigersaurus*, *Diplodocus* and *Brachiosaurus*, and *Brachiosaurus* and *Camarasaurus* were indistinguishable from each other (Table 5). This suggests that *Diplodocus* and *Nigersaurus* were both primarily browsing on similar foods, although *Diplodocus* may also have incorporated a small component of hard foods in its diet; such a diet may explain the observed similarity to *Brachiosaurus*. A diet heavy on silica-accumulating plants may also have caused this; one such plant, *Equisetum*, is known from the Morrison Formation, and has previously been suggested to form a component of the diet of *Diplodocus* [80]. *Dicraeosaurus*, which differed significantly in scratch breadth with all other study taxa, was potentially also a mixed feeder.

**Table 5:** Results of pairwise comparisons (after [102]) following a Kruskal-Wallis test on samples of scratch breadth.

	*Brachiosaurus*	*Camarasaurus*	*Dicraeosaurus*	*Diplodocus*	*Nigersaurus*
*Brachiosaurus*	—	**1.000**	0.00	**0.216**	0.000
*Camarasaurus*	**1.000**	—	0.00	0.000	0.000
*Dicraeosaurus*	0.00	0.00	—	0.000	0.000
*Diplodocus*	**0.216**	0.000	0.000	—	**0.164**
*Nigersaurus*	0.000	0.000	0.000	**0.164**	—

Results in bold indicate cases where the null hypothesis (distributions in each sample are the same) cannot be rejected. Putative high browsers (*Brachiosaurus* and *Camarasaurus*) cannot be distinguished based on scratch breadth. *Dicraeosaurus* is distinguishable from all others. *Diplodocus* and *Nigersaurus* cannot be distinguished.

The greater orientational consistency in *Diplodocus* and *Nigersaurus* suggests that these animals were biting through softer stems, consistent with a hypothesis of browsing on herbaceous plants, such as low-growing ferns. Conversely, the low consistency of scratches in *Dicraeosaurus* is indicative of harder, more brittle foods, possibly including the shearing of woody stems. The significantly coarser scratches and larger pits/gouges observed *Dicraeosaurus* also suggest a diet including coarser plants and a large proportion of larger particles than those ingested by *Diplodocus* and *Nigersaurus*, and probably *Apatosaurus* and *Rebbachisaurus* as well. The larger particles in question may have been hard objects such as seeds; an abundance of such high-quality foodstuffs in the diet indicates selective browsing behavior [118]. *Brachiosaurus* and *Camarasaurus* have relatively high orientational consistencies despite the common occurrence of cross-scratches. In those taxa, the higher than expected orientational consistency is likely a function of an interlocking dentition and precise occlusion, two features not seen in diplodocoids.

Pit/gouge size was also found to differ significantly between several of the taxa (H = 229.386, df = 2, P = 0.000). Pairwise comparisons found significant differences in pit/gouge area between *Diplodocus* and all others and *Nigersaurus* and all others, but not between *Brachiosaurus*, *Camarasaurus*, and *Dicraeosaurus* (Table 6). Pit/gouge size appears to be able to distinguish between selective and non-selective (or less selective) browsers, although *Diplodocus* and *Nigersaurus*, both potentially non-selective browsers as predicted by snout shape, had statistically distinguishable pit/gouge size distributions as well. Average pit size in the two taxa is quite similar (34.6 µm^2^ in *Nigersaurus*, 22.7 µm^2^ in *Diplodocus*), however, and the next most-similar is substantially larger (*Camarasaurus*, at 88.4 µm^2^), suggesting that the perceived difference between pit/gouge size in *Diplodocus* and *Nigersaurus* may not be functionally significant.

**Table 6:** Results of pairwise comparisons (after [102]) following a Kruskal-Wallis test on samples of pit area.

	*Brachiosaurus*	*Camarasaurus*	*Dicraeosaurus*	*Diplodocus*	*Nigersaurus*
*Brachiosaurus*	—	**1.000**	**1.000**	0.000	0.000
*Camarasaurus*	**1.000**	—	**1.000**	0.000	0.000
*Dicraeosaurus*	**1.000**	**1.000**	—	0.000	0.000
*Diplodocus*	0.000	0.000	0.000	—	0.005
*Nigersaurus*	0.000	0.000	0.000	0.005	—

Results in bold indicate cases where the null hypothesis (distributions in each sample are the same) cannot be rejected. None of the taxa hypothesized to be selective browsers can be distinguished statistically from each other.

In addition to their significantly larger size, the features recovered from *Camarasaurus*, *Dicraeosaurus* and *Brachiosaurus* are notably more oblate and gouge-like than the subcircular features recovered from the other taxa. Although the meaning of this shape difference is uncertain, it may be related to physical differences in the particles causing the wear, or to the orientation of the force compressing the particle into the tooth (e.g., meeting the enamel edge obliquely vs. orthogonally). In either case, the shape difference suggests some significant difference in diet or behavior between *Dicraeosaurus* and the square-snouted diplodocoids, as has been previously suggested [30].

### Comparison with previous results

#### Diplodocus

Fiorillo [22], [23], Calvo [26], and Upchurch and Barrett [30] previously examined wear features on the teeth of *Diplodocus*. All studies agree on the dominance of fine, subparallel scratches oriented generally along the labiolingual axis. Neither Fiorillo [22], [23] nor Calvo [26] recovered pits or gouges. Upchurch and Barrett [30] did recover large pits, although these were considered larger than those normally produced by grit or phytoliths. These features appear to have been recovered from the dentine of a heavily worn tooth, however [30], where larger features may be expected. The lack of pits led Fiorillo [22], [23] and Calvo [26] to suggest an upper-canopy browsing behavior for *Diplodocus*, which is counter to much of the evidence for ground-level browsing presented more recently [10], [27], [28], [30]. High browsing was also inferred as a possible feeding mechanism for *Diplodocus* by Upchurch and Barrett [30], based on macrowear features.

The absence of pits/gouges in most previous studies is somewhat perplexing, given their relative abundance in all teeth examined in this study. Further confusion comes from the overlap in sample between a number of studies: this study, the study of Upchurch and Barrett [30], and the studies of Fiorillo [22], [23] all examine the same specimen of *Diplodocus* (CM 11161), but obtain a spectrum of results ranging from a majority of pits (this study), to some pits [30], to no pits [22], [23]. As a result, it is unlikely that such biological factors as seasonality are responsible for the differences, at least in those samples that directly overlap. The different patterns of features may be a result of methodology, given the reliance of previous studies on SEM images (at extremely high magnification) as opposed to the use of low-magnification microscopy here. It may also be the result of sample size, as all studies (including the current study) examined relatively few teeth per taxon compared to studies of mammals. The ubiquity of pits in all teeth examined here, their similarity in form among broad-snouted taxa, and their difference in form in the round-snouted *Dicraeosaurus*, however, all reinforce the validity of the interpretation presented here.

#### Nigersaurus

Sereno et al. [10] examined microwear features on the teeth of *Nigersaurus*. The results of that study are essentially identical to those presented here, although this study identified a significantly larger number of small pits. Both Sereno et al. [10] and this work infer a diet based on ground-level browsing of herbaceous plant materials based on the consistent labiolingual orientation of fine scratches on the labial surface of the enamel and a high pit/scratch ratio.

### Leaf stripping behavior?

Ryan [75] suggested that some features of incisor microwear in *Gorilla*, *Pan*, and *Papio* were indicative of leaf-stripping behavior: polished surfaces and a preponderance of subparallel, fine scratches on the apical surface of the incisors. However, the proportion of features (scratches and pits) in the microwear assemblages reported here for sauropods differ from wear produced by leaf-stripping. In all but one sample (left premaxillary tooth 1 of *Diplodocus*) from the occlusal surface of sauropod teeth, pits outnumber scratches significantly, whereas scratches substantially outnumber pits in each of the samples reported by Ryan [75]. This latter result was suggested to have been a consequence of repeated drawing of plant materials over the enamel surface. In leaf stripping behavior, grit causes striations rather than compressional features (i.e., pits) as seen in wear caused by a bite. Because of the high proportion of pits in the sauropod microwear sampled, leaf stripping behavior is considered to be less plausible than ground-level browsing behavior, although it is noted that leaf stripping and ground-level browsing do leave superficially similar traces on incisiform teeth.

### Non-facet microwear features

Features recorded from the non-occlusal surfaces of presumptive ground-level browsing taxa such as *Diplodocus* and *Nigersaurus* differ from the features recorded from facets in both taxa. Specifically, larger features are recovered, and those features (particularly scratches) are in a different orientation relative to the tooth, nearly orthogonal to the long axis of the tooth rather than subparallel to it. Because these features are located outside the occlusal surface, it is highly unlikely that they represent wear formed during the bite stroke. The implication, therefore, is that the wear was caused by nearby vegetation, which in turn suggests a dense (perhaps sward-like) growth form for the food resource in question. The orientation of the wear at nearly 90° to the occlusal features may have been the result of vegetation scraping against the labial margin of the upper dentition as the head and neck are moved laterally to obtain the next bite; this would fit with the interpretation of some authors [27], [28] of the long neck as a means to increase the feeding envelope without moving the body. These features may also be have resulted from incidental contact in leaf-stripping behavior, although as noted above that behavior is considered to be less likely. In either case, root causes of such features are poorly constrained and non-facet microwear is unlikely to serve as evidence for feeding behavior at this point in time. Such features were not recorded from the selectively browsing *Dicraeosaurus*.

### Feeding Behavior: Summary

Snout shape and microwear indices suggest the presence of both a ground-level, nonselective browsing behavior and a mid-height (above 1 m), selective browsing behavior in diplodocoid sauropods. Here, feeding behavior is examined in relation to body size, phylogeny, and paleoecology, to determine the influence of each on behavior in diplodocoid sauropods.

#### Body size and feeding behavior

Among flagellicaudatans (the group containing Diplodocidae and Dicraeosauridae), selectively browsing taxa had smaller skulls, and were smaller overall, than non-selective browsers [34], [119]–[121]. The rebbachisaurid *Nigersaurus*, however, was the most specialized non-selective browser sampled and was of similar size to *Dicraeosaurus* and *Tornieria*. Although this rules out the hypothesis that feeding behavior was entirely size-dependent, it does not necessarily mean that there is no relationship between size and behavior. It is possible that above 10–12 meters in body length, selective browsing behavior became untenable as a feeding mode (although this restriction does not appear to apply to upper-canopy feeders like *Brachiosaurus* and *Camarasaurus*); non-selective browsing behavior was clearly effective even at small (by sauropod standards) body sizes. Other dinosaurs hypothesized to have been ground- and mid-height selective browsers, such as ceratopsians [47], heterodontosaurs [82], lambeosaurines [46], and stegosaurs [122] also tended to be small in comparison to sauropods, and even the largest rarely exceeded 12 m in body length [47], [123]–[126]. It is possible that above this size, handling and forage time for selective browsing exceeded some metabolic threshold when an animal is limited to lower-canopy browse. Selective vs. nonselective browsing strategies do generally scale with body size in mammals [127], although the degree to which this analogy would be expected to hold for dinosaurs is uncertain. The relationship in mammals is based primarily on increased retention time as a function of increased body (and gut) size in large mammals [128] and relatively increased metabolic requirements in smaller mammals [129]; sauropods and other herbivorous dinosaurs often far surpassed the body sizes of even very large mammals [130], however, and estimates of metabolic rate in dinosaurs are still fraught with uncertainty [131]–[134]. However, the relationship between body size and the relative rarity of high-quality forage in most ecosystems and its associated foraging cost is also documented in mammals [129], [135], [136], providing some evidence for an upper size limit on selective browsing in sauropods. Although macronarian sauropods successfully grew large while selectively browsing in the upper canopy, they also had exclusive dominion over those heights as no other clade of dinosaurian herbivore was able to access those resources [137].

#### Phylogenetic signal

**Figure 7:**
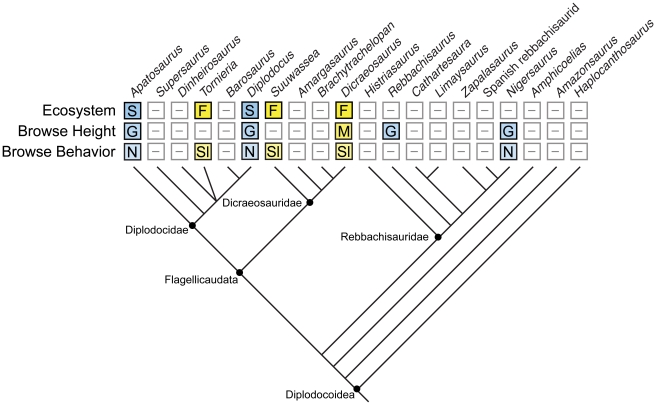
Phylogeny of diplodocoid sauropods (modified from [**103**]), with ecosystem, inferred browse height, and inferred browse behavior plotted above terminals. Data suggest that ground-height, non-selective browsing evolved in open, savanna-like environments, whereas selective, mid-height browsing was most common in diplodocoids living in closed environments dominated by mid- and upper-canopy browse. Blue tones indicate data suggestive of ground-height, non-selective browsing; yellow tones indicate data suggestive of mid-height, selective browsing. Inferences for which insufficient data exists are represented in 50% grey tones. Abbreviations: S, savanna type ecosystem; F, forested ecosystem; G, ground-height browser; M, mid-height browser; N, nonselective browser; Sl, selective browser.

Within individual diplodocoid clades, feeding behavior was reasonably consistent, although snout shape can be determined for only one rebbachisaurid (*Nigersaurus*; Fig. 7). However, the diplodocid *Tornieria* has microwear features and a snout shape most similar to those of the dicraeosaurids *Dicraeosaurus* and *Suuwassea*. Because *Tornieria* is a relatively derived diplodocid [103], [121], it appears that the feeding behavior inferred for this taxon is an independent derivation within this lineage, rather than a retained plesiomorphic behavior. This in turn suggests that the behavior was not strictly governed by inheritance, and that some plasticity was possible.

The ancestral condition for snout shape in diplodocoids is difficult to determine. Although both diplodocids and rebbachisaurids appear to have square snouts, there is no overwhelming evidence to suggest that this is the original condition for the group as a whole. The ancestral state in Flagellicaudata is equivocal: basal diplodocids have square snouts and basal dicraeosaurids have round snouts. Furthermore, the only rebbachisaurid for which snout shape can be determined (*Nigersaurus*), is a highly derived taxon that likely does not represent the basal condition for the group. Juvenile *Diplodocus* have narrow snouts [32], which may suggest that the square-snouted condition seen in adults is derived from an ancestral round-snoutedness. Outgroup taxa (e.g., macronarians, basal eusauropods) are unequivocally round-snouted, but where, when, and how many times the transition to square-snoutedness occurred in diplodocoidea cannot be said with certainty. If square-snoutedness is taken as the ancestral diplodocoid condition, one origination (at the base of Diplodocoidea) and two reversals (one at the base of Dicraeosauridae, once in *Tornieria*) are required (three evolutionary steps). If round-snoutedness is basal for sauropods, then at least two originations (once at the base of Diplodocidae and once within Rebbachisauridae) and one reversal (*Tornieria*) are required (also three steps).

#### Environmental signal

Similar morphological plasticity in other dinosaurian groups (e.g., hadrosaurids) has typically been interpreted in relation to behavior, although often behavior and morphological divergence are correlated with phylogeny [46], and it can be difficult to tease apart the influences of phylogeny and behavior on morphology. However, as noted above, phylogeny is not a perfect explanation for the diversity of snout shapes seen in diplodocoids. It is likely, then, that this morphological variability was driven in larger part by the browse flora available to these animals.

Herbivory imposes two major constraints on behavior: foraging time and digestive time [135]. Digestive time is a function of the length of the digestive tract and may be loosely interpreted from body size, although soft tissue structures (e.g., the rumen), which are not typically preserved in the fossil record, can substantially impact retention time [80], [138]. Foraging time, however, can be inferred from skeletal evidence based on models of intake rate. Intake rate has a major influence on feeding behavior in modern herbivores [139]; which is to say that an organism will attempt to maximize intake rate in a given environment. Broad or square snouts have been demonstrated to maximize intake rate in non-selectively browsing mammals [40]; a broad snout in selective herbivores decreases intake rate by increasing handling time and minimizing effective bite mass cropped [140].

Variation in forage quality may also have had some influence on behavior. Two hypotheses in particular, the forage abundance hypothesis (FAH) [141] and the selective quality hypothesis (SQH) [142], might be useful in explaining a relationship between ecology and behavior in these sauropods. The FAH suggests that when resources are perceived to be abundant, animals may choose to be more selective; grazing behavior increases when resource levels are low. In this hypothesis, forage abundance is the controlling factor. SQH, conversely, predicts that herbivores are actually less selective when high quality resources are plentiful and homogenously distributed. Under SQH, food quality is the key variable. If the regional ecology can be reconstructed for sauropods, support for one or both of these hypotheses in the feeding behavior may be found.

Although the ecological conditions of Niger during the Aptian-Albian are largely unknown (particularly with regard to flora), the paleoecology of the Late Jurassic Morrison and Tendaguru formations, where the remaining five taxa have been found, is better understood.

The Morrison Formation of North America is dominated by diplodocoid sauropods, in particular the square-snouted diplodocids. Diplodocids (primarily *Apatosaurus* and *Diplodocus*) are both the most common sauropod fossils found in these beds and the most widespread, occurring in more localities than any other clade [143] (n.b., the macronarian *Camarasaurus* is the most numerous single genus of dinosaur [144]). Only a single round-snouted diplodocoid taxon (*Suuwassea*) is known from the Morrison Formation.

Plant fossils from the Morrison include a wide diversity of conifers, ginkophytes, podocarpaceans, ferns, cheirolepidiaceans, and horsetails [145]–[147]. Most recent work suggests that much of the Morrison Formation was an arid to semi-arid savanna-like environment, dominated by ground-height herbaceous browse (e.g., ferns, bryophytes) and low- to mid-height woody shrubs [147], [148]. Taller browse (primarily conifers; e.g., *Pagiophyllum*, *Podozamites*) was restricted to areas near watercourses and isolated pockets, such as that preserved by the Salt Wash member [146]–[148]. It is in these isolated pockets that high-browsing sauropods like *Brachiosaurus altithorax* are found [148].

Low browse in the southern Morrison, particularly the ferns and small trees, appears to have been highly nutritious and digestible, on par with extant browse [80]. The abundance of high quality, broadly distributed low browse and the appearance of multiple lineages of non-selectively browsing sauropods fit well with the predictions of the SQH and suggest a relationship between ecology and behavior in the southern Morrison Formation.

The northern end of the Morrison Formation (e.g., Montana) has been recognized for its unusual sauropod fauna, composed of smaller adults and more juveniles than is typical of more southern localities [120], [149]. Additionally, dicraeosaurids such as *Suuwassea* and MOR 592 are known exclusively from Montana [120], [150]. Paleoenvironmental reconstructions of the northern Morrison Formation are also quite different from those of the southern localities. This region was likely to have been a wetter environment than the southern Morrison Formation [107], [147], [151]. Morrison Formation sediments found near central Montana are believed to have been deposited in mires, coal swamps and/or peat bogs, and associated riparian environments [147]. Forested habitats have previously been thought to lead to a greater reliance on selective browsing in other dinosaurs [46] and in modern mammals [39], [135]. It is probable that the restriction of round-snouted, selectively browsing dicraeosaurids to this type of environment in the Morrison Formation indicates a similar constraint on sauropod dinosaurs. The less homogenous environments of the northern Morrison may have resulted in patches of highly nutritious vegetation, such as *Equisetum* and the conifer *Araucaria* [80]; the increase in selectivity when high-quality resources are restricted matches the predictions of SQH. The relatively high concentration of juvenile diplodocoids in the northern Morrison Formation, with their narrow snouts [32], is also consistent with this interpretation.

Unlike that of the Morrison, the sauropod fauna of Tendaguru is made up exclusively of round-snouted taxa, including *Brachiosaurus*, *Dicraeosaurus*, and *Tornieria*. The African *Brachiosaurus* taxon is a much more important component of the fauna compared to its American relative, particularly in the Middle Saurian Beds, where it is the most common sauropod found [152]. The most common fossil sauropod in the Upper Tendaguru is the diplodocid *Tornieria*, which is rare in the Middle Saurian Beds but common elsewhere [152]. *Dicraeosaurus* is a minor component of all dinosaur-bearing layers [152].

The sediments preserved in the Tendaguru Formation encompass both strictly terrestrial uplands and tidal flats/coastal regions, but the latter appear to have been poorly vegetated [153]. In contrast, the uplands seem to have been heavily vegetated by conifer forests, including the very tall araucarians that would have provided a food source for high-browsing sauropods [153]. Also present were evergreen shrubs and small (<25 m) trees in the family Podocarpaceae [153]. Minor components of the flora included cycads and ginkophyte trees. Ferns were exceptionally rare, and only two varieties, either *Dicroidium* or *Pachypteris*-type, have been reported from Tendaguru [153]. The uplands of Tendaguru, therefore, were a heterogenous mix of upper-canopy browse dominated by conifers (Cheirolepidiaceae and Araucariaceae) and mid-height woody browse (Podocarpaceae, cycads); comparatively little ground-level bulk forage (ferns) would have been present.

No ground-height, non-selectively browsing sauropod dinosaurs have been recovered from Tendaguru. The sauropods that have been found there are exclusively mid-height (*Dicraeosaurus*, *Tornieria*) or upper canopy (*Brachiosaurus*) feeders, which corresponds well with the recovered vegetation, which is dominated by woody browse. Although cycads and podocarpacean evergreens would have been abundant mid-height browse, they are substantially less nutritious than the ferns, horsetails, and other low browse plants common to the southern Morrison Formation [80]. Here, where high-quality food resources are limited, SQH predicts an increase in selectivity. The lack of evidence for large bodied, non-selective browsers in Tendaguru fits well with this prediction.

#### Summary

Forested habitats that are linked with riparian environments are also associated with round-snouted sauropods in both the Morrison (*Brachiosaurus altithorax*, *Camarasaurus*, *Suuwassea*) and Tendaguru (*Brachiosaurus brancai*, *Dicraeosaurus*, *Tornieria*) Formations. Square-snouted sauropods (*Apatosaurus*, *Diplodocus*) are found in the open, savanna-like environment proposed for the southern Morrison Formation, although the round-snouted *Camarasaurus* is also found in these beds. Evidence suggests that the riparian environments cutting through the savanna-type environments of the Morrison had substantial tree coverage [148], which could explain the presence of *Camarasaurus* in those regions. The selective quality hypothesis predicts non-selective browsing when high-quality resources are abundant and broadly distributed and selective browsing when high-quality resources are restricted [142]; both predictions match the inferences of diplodocoid feeding strategy and floral ecology made for the Morrison and Tendaguru Formations. The general robustness of the relationship between diplodocoid anatomy and paleoecology suggests that square-snoutedness is linked with ground-height, non-selective browsing, and round-snoutedness is associated with mid- to upper-canopy selective browsing (Fig. 7).

### Conclusions

Hypotheses of feeding behaviors typical of modern mammalian herbivores (e.g., non-selective and selective browsing) are supported for diplodocoid sauropods using evidence from snout shape and dental microwear. Snout shapes in diplodocoids include both rounded and square snouts, similar to those seen in hadrosaurid dinosaurs. Square snouts have been correlated with non-selective feeding behavior in modern and extinct mammals and in hadrosaurine dinosaurs, whereas round snouts correlate with selective browsing behaviors in those taxa. Dental microwear features indicative of ground-height browsing on herbaceous plants correspond with square snouts in diplodocoid sauropods; microwear features suggestive of mid-height browsing on brittle, potentially woody plants correspond with round snouts.

There is a potential correspondence between body size and feeding behavior in diplodocoids: above 12–15 m body length, diplodocoids are exclusively non-selective, ground-height browsers; small diplodocoids include both selective and non-selective browsers, however. There is probably not a strong phylogenetic signal to morphology and behavior, although most diplodocids (except *Tornieria*) and rebbachisaurids were ground-height browsers, and dicraeosaurids (and *Tornieria*) were mid-height-browsers, although potentially restricted to the lower portion of that feeding zone. Feeding behavior corresponds well to environmental associations, such that closed environments dominated by upper-canopy browse lacked the ground-height, nonselective browsers that dominated open environments.

## Supporting Information

Table S1 Material examined for cranial reconstructions.
(DOC)

Table S2 Teeth examined for microwear features. Teeth without position indicated were found isolated.
(DOC)

Table S3 Complete list of microwear features (including dimensions, area, and type) for all teeth examined.
(XLSX)

Table S4 Snout shape scores (PMI) for the hadrosaurids examined.
(DOC)
