# Supplementary material for: Inferences of Diplodocoid (Sauropoda: Dinosauria) Feeding Behavior from Snout Shape and Microwear Analyses
Source: PLoS One. 2011 Apr 6;6(4):e18304. doi: 10.1371/journal.pone.0018304 (PMC3071828; doi:10.1371/journal.pone.0018304)
Supplement: Table S1 — Material examined for cranial reconstructions. (DOC) [file pone.0018304.s001.doc]

**Table S1.** Material examined for cranial reconstructions

| Taxon | Material Examined |
| --- | --- |
| *Apatosaurus* | CM 11162, CMC VP7180 |
| *Dicraeosaurus* | MB.R. 2336, MB.R. 2337, MB.R. 2338, MB.R. 2339, MB.R. 2340, MB.R. 2372, MB.R. 2378, MB.R. 2379 |
| *Diplodocus* | AMNH 969, CM 11161, USNM 2672, USNM 2673 |
| *Nigersaurus* | MNN GAD 512 |
| *Suuwassea* | ANSP 2112 |
| *Tornieria* | MB.R. 2343, MB.R. 2346, MB.R. 2348, MB.R. 2374, MB.R. 2387, MB.R. 2388 |
