# Supplementary material for: Inferences of Diplodocoid (Sauropoda: Dinosauria) Feeding Behavior from Snout Shape and Microwear Analyses
Source: PLoS One. 2011 Apr 6;6(4):e18304. doi: 10.1371/journal.pone.0018304 (PMC3071828; doi:10.1371/journal.pone.0018304)
Supplement: Table S2 — Teeth examined for microwear features. Teeth without position indicated were found isolated. (DOC) [file pone.0018304.s002.doc]

**Table S2.** Teeth examined for microwear features. Teeth without position indicated were found isolated.

| Taxon | Material Examined |
| --- | --- |
| *Apatosaurus* | CMC VP 7180: right maxillary tooth 4, right dentary tooth 2 |
| *Dicraeosaurus* | MB.R.2195:one tooth; MB.R.2197:one tooth; MB.R.2204:one tooth |
| *Diplodocus* | CM 11161: left premaxillary teeth 1–4, left maxillary teeth 1, 4, right premaxillary teeth 2, 3, right maxillary tooth 1; USNM 2672: left dentary tooth 1, left maxillary teeth 5, 6, right premaxillary tooth 2, right maxillary teeth 2, 5–7; USNM 2673: six loose teeth |
| *Limaysaurus* | MUCPv-205: two teeth |
| *Nigersaurus* | MNN GAD 512: G2: one tooth; G89: three teeth; G110: three teeth |
| *Rebbachisaurus* | MNHN 1524a: one tooth |
| *Suuwassea* | ANSP 2112: one tooth |
| *Tornieria* | MB.R.2193: right maxillary teeth 1, 2 |
| *Camarasaurus* | CM 11388; UUVP 1949; UUVP 1950; UUVP 3971; UUVP 3986 |
| *Brachiosaurus* | MB.R. 2181: teeth 2,3,4, and 9; MB.R.2190 |
