# Supplementary material for: Inferences of Diplodocoid (Sauropoda: Dinosauria) Feeding Behavior from Snout Shape and Microwear Analyses
Source: PLoS One. 2011 Apr 6;6(4):e18304. doi: 10.1371/journal.pone.0018304 (PMC3071828; doi:10.1371/journal.pone.0018304)
Supplement: Table S4 — Snout shape scores (PMI) for the hadrosaurids examined. (DOC) [file pone.0018304.s004.doc]

**Table S4**. Snout shape scores (PMI) for the hadrosaurids examined.

| Taxon | Specimen/Reference | PMI |
| --- | --- | --- |
| *Anatotitan copei* | AMNH 5730; [109] | 93 |
| *Edmontosaurus regalis* | NMC 2288; [109] | 84 |
| *Maiasaura peeblesorum* | PU 22405; [111] | 84 |
| *Prosaurolophus maximus* | AMNH 5386; [112] | 79 |
| *Saurolophus osborni* | AMNH 5220; [113] | 81 |
| *Velafrons coahuilensis* | CPC-59; [114] | 85 |
| *Lambeosaurus sp.* | ROM 758 | 74 |
| *Hypacrosaurus altispinus* | ROM 702 | 76 |
| *Corythosaurus sp.* | NMC 34825 | 80 |
